# Annular Flow in the Upper Esophageal Sphincter Demonstrated with Dynamic 320-row Area Detector Computed Tomography

**DOI:** 10.1007/s00455-020-10241-9

**Published:** 2021-01-28

**Authors:** Yoko Inamoto, Eiichi Saitoh, Jeffrey B. Palmer

**Affiliations:** 1 Faculty of Rehabilitation, School of Health Sciences, Fujita Health University, 1-98 Dengakugakubo, Kutsukake, Toyoake, Aichi 470-1192 Japan; 2 Department of Rehabilitation Medicine I, School of Medicine, Fujita Health University, Toyoake, Aichi, Japan; 3 Department of Physical Medicine and Rehabilitation, Johns Hopkins University, Baltimore, MD USA

## Abstract

Understanding bolus flow patterns in swallowing (rheology, the study of flow) is fundamental to assessment and treatment of dysphagia. These patterns are complex and poorly understood. A liquid swallow is typically biphasic, including air, so the actual bolus has both liquid and gas phases. We report a novel observation of annular two-phase flow (a ring of liquid around a core of air) as thin liquids passed through the upper esophageal sphincter (UES). Dynamic CT was performed on 27 healthy asymptomatic volunteers swallowing liquid barium in a semi-reclining position. Each subject swallowed 3, 10, and 20 ml of either thin (14 subjects) or thick liquid (13 subjects). Sagittal and axial images were analyzed. Flow patterns in the UES were assessed on cross-sectional images. Annular flow was seen in the majority of subjects with thin liquid but few with thick liquid swallows. The percentage of Annular flow during UES opening was 3 ml 58%, 10 ml 58%, 20 ml 56% in thin and 3 ml 0%, 10 ml 4%, 20 ml 1% in thick. Annular flow was usually observed from the second or third frames after onset of UES opening. The other pattern, Plug flow was seldom seen with thin but was typical with thick liquid swallows. Annular flow was the most common pattern for thin liquids (but not thick liquids) passing through the UES. Annular flow has been defined as a liquid continuum adjacent to the channel wall with a gas continuum (core) in the center of the channel. The two regions are demarcated by a gas–liquid interface. Annular flow is typical for two-phase gas–liquid flow in a vertical or inclined channel. It results from the interaction of viscosity with cohesive and adhesive forces in the two phases. We infer that the difference in flow pattern between thin liquid–air and thick liquid–air boluses resulted from the differing magnitudes of viscous forces.

## Introduction/Background

Upper esophageal sphincter (UES) is a complex muscular-skeletal-epithelial structure. It contracts between swallows and relaxes during swallowing. Multifactorial elements are involved in the mechanism of UES opening, including sphincter relaxation, traction by anterosuperior laryngeal elevation, and forces within the bolus [1–3]. Among them, forces within the bolus represents the bolus driving force from pharynx to esophagus. Intrabolus pressure (IBP) is also an element of UES opening and has been utilized as a clinical marker for bolus transport [4]. It has been reported that the bolus head has the lower pressure and the bolus tail has stronger pressure. This pressure gradient is critical to move the bolus from high to low pressure [5]. An increased intrabolus pressure gradient identifies reduced UES opening [6].

Although, many studies using manometry reported the importance of assessing intrabolus pressure, which is thought to be a predictor of swallowing dysfunction, bolus flow patterns through UES are little known. This is because most previous studies observed the bolus flow during swallowing using videofluoroscopy, which provides two-dimensional imaging. It reveals lateral and anteroposterior views of bolus movement from oral to esophagus, but not the transverse (cross-sectional superior-inferior) view. Understanding bolus flow patterns in swallowing (rheology, the study of flow) is fundamental to assessment and treatment of dysphagia. It is an important element to promote a safe and efficient swallow.

By the observation of UES cross-section using 3D dynamic computed tomography (CT), a novel finding of flow which is a ring of liquid around a core of air (annular flow) was noted when liquid was passing through the upper esophageal sphincter (UES). It seemed not to be common when thick liquid was passing through UES. Therefore, we aimed to study the bolus flow pattern (flow regime) with different bolus consistencies and volumes using 3D dynamic CT. A fluid bolus is typically biphasic because it includes both liquid and gas (air) [7]. The flow of biphasic liquids is governed by the interplay of several forces, including viscosity, cohesion, adhesion, internal friction, and surface tension. We hypothesize that the observed bolus flow patterns in the upper esophageal sphincter are due to the interplay of viscosity with cohesive and adhesive forces.

## Material and Methods

Dynamic CT was performed on 29 healthy asymptomatic volunteers swallowing liquid barium in a semi-reclining position at a 45-degree angle. Each subject swallowed 3, 10, and 20 ml of thin (14 subjects, 34.4 ± 5.0 years) or honey-thick liquid (15 subjects, 37.8 ± 7.6 years) according to the command of examiner. Both thin (1.17 mPa) and honey-thick (1700 mPa) liquids were mixed with Barium (5%w/v). Scanning was performed with a 320-row area detector CT (320-ADCT, Canon Medical Systems, Tochigi, Japan). The scanning duration was 3.15 s. Scanning parameters were as follows; scanning range = 160 mm from skull base to the cervical esophagus, field of view = 240 mm, tube voltage/current = 120 kV, 40 mA. The effective dose was estimated 3.24 mSv for three swallows [8]. All subjects gave informed consent for the study based on protocols approved by the Institutional Review Board.

**Fig. 1:**
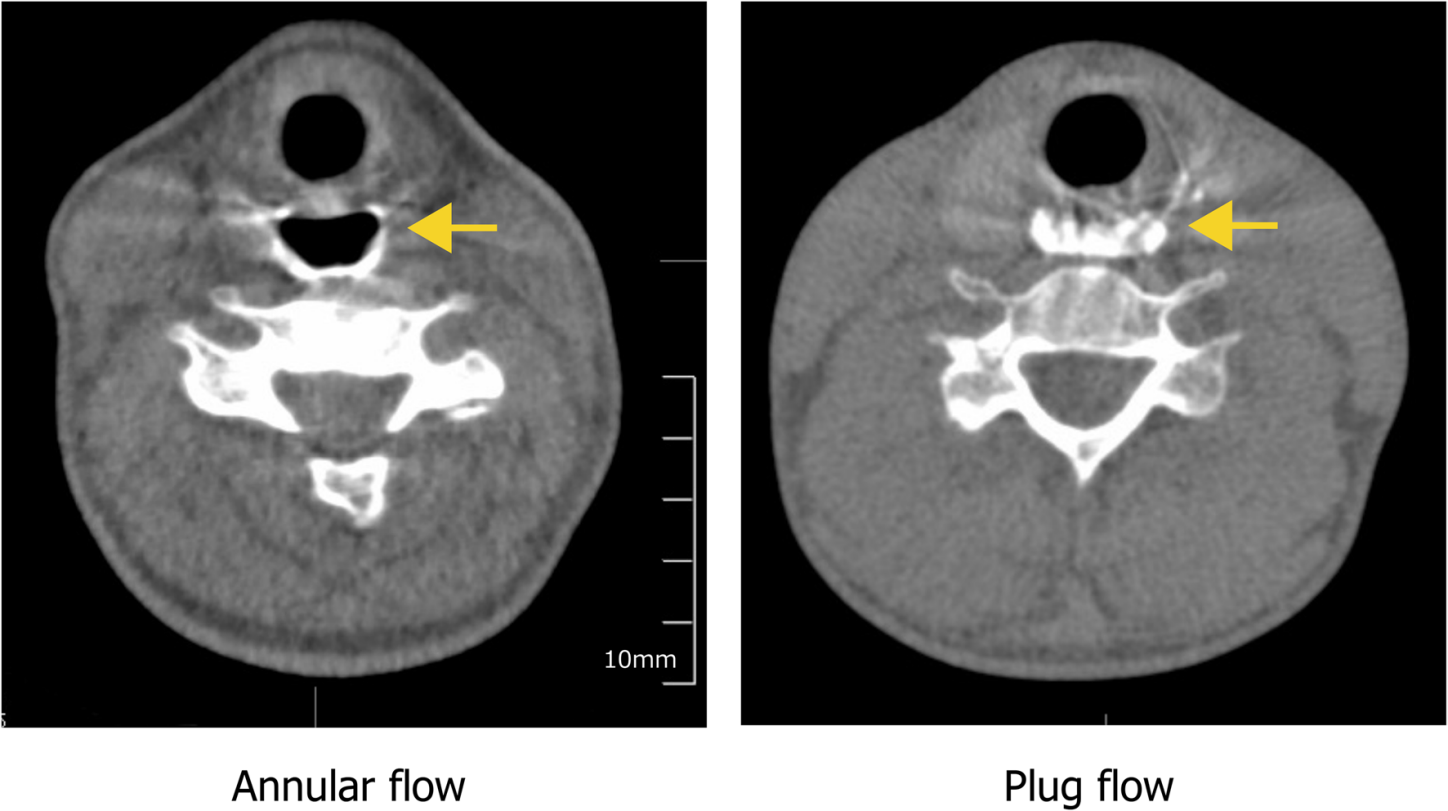
Definition of Annular flow and Plug flow. Annular flow was defined as the annulus of liquid barium lined the wall of the UES and surrounded an inner core of air flowing. Plug low was defined as the lumen of the UES was entirely filled by the liquid phase and no airflow

Multi-planar reconstruction (MPR) images and 3D-CT images were created at an interval of 0.10 s (10 images/s) from the 0.5 mm slice thickness axial slices using scanner software. Sagittal and axial views of MPR images were analyzed. Flow patterns in the UES were assessed on cross-sectional images and classified as either Annular flow or Plug flow (Fig. 1) by two independent raters, who were proficient in diagnostic imaging using 320-ADCT. Differences were resolved by discussion. Annular flow was recognized as an annulus (ring) of liquid barium lining the wall of the UES and surrounding an inner core of air (both flowing toward the esophagus). Plug flow was recognized when the gas and liquid phases alternated. Thus, on some images the lumen of the UES was entirely filled by the liquid phase with no visible air. The UES was identified on axial sections at the level of lower end of the inferior horn of thyroid cartilage using the same method used in previous study [9]. Mann–Whitney U Test was used to compare the relative frequencies of Annular flow between the two consistencies. The kappa coefficient was calculated to estimate the interrater reliability.

## Results

**Fig. 2:**
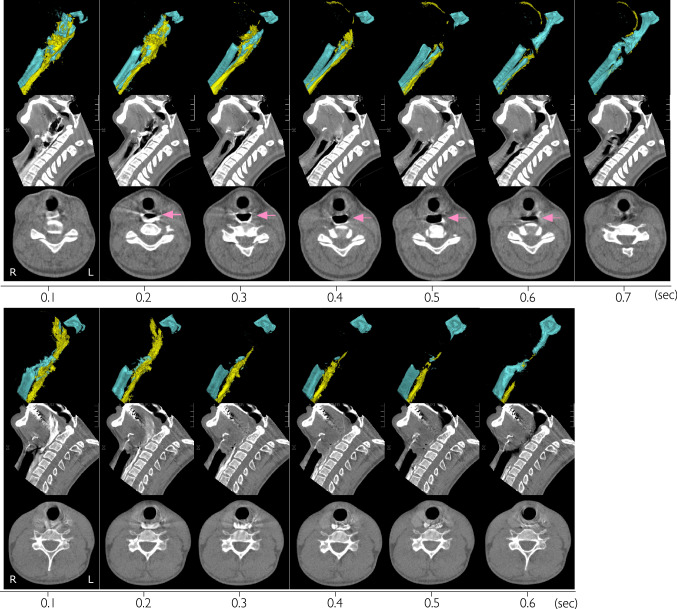
Lateral, mid-sagittal, axial views (upper, middle, and lower rows, respectively) of 3D-CT images during swallows of thin and thick liquid in representative subjects. Upper three rows are the images of thin liquid swallowing in a representative subject (37y, female). Lower three rows are the images of honey liquid swallowing in a representative subject (24y, male). In both upper and lower parts, first line is lateral 3D-CT images (yellow: bolus, blue: air column surface), second line is mid-sagittal sections, and third line is axial sections of UES. Arrows show annular flow. L: left R: right

**Fig. 3:**
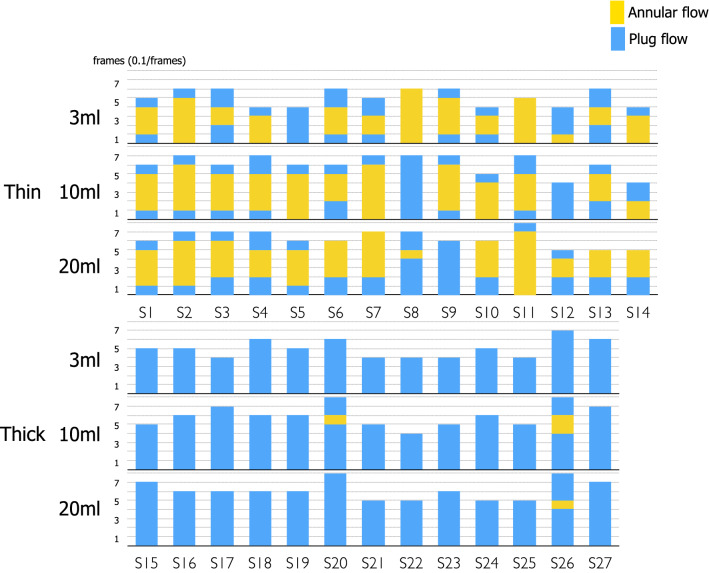
Images showing Annular flow during UES opening in swallows of thin and thick liquid in each subject. Upper row is 3 ml, middle row is 10 ml, and lower row is 20 ml swallows with both thin and thick liquids. *Y* axis is the frame number (0.1 s/frame) from onset of UES opening (duration of UES opening). Yellow shows Annular flow and blue shows Plug flow. Annular flow was typically observed within the first three frames after onset of UES opening with thin liquids

**Fig. 4:**
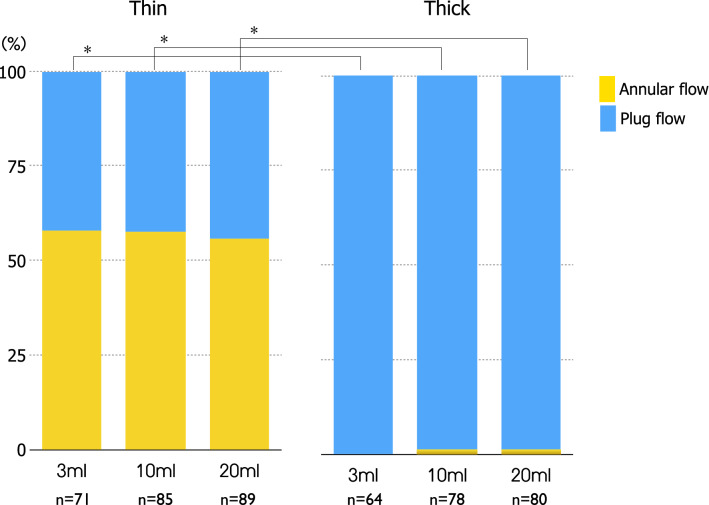
Relative frequencies of Annular flow and Plug flow in swallows of thin and thick liquid. Annular flow was seen during UES opening in 58%, 58%, 56% of 3, 10, 20 ml swallows, respectively with thin liquid and 0%, 4%, 1% in 3, 10, 20 ml swallows, respectively with honey-thick liquid. The percentage of Annular flow was significantly higher in all bolus sizes with thin than with thick liquid swallows (*p *< 0.001). Yellow shows Annular flow and blue shows Plug flow

The average duration of UES opening in 3 ml, 10 ml, 20 ml swallows were 0.51 ± 0.09 s, 0.61 ± 0.11 s, 0.63 ± 0.09 s (mean ± SD) for thin liquids and 0.52 ± 0.09 s, 0.62 ± 0.09 s, 0.64 ± 0.09 s for thick liquids. There was no significant difference between thin and thick in the duration of UES opening. Two kinds of bolus flow patterns, Annular and Plug flows were observed on cross-sectional views of the upper esophageal sphincter (UES) in swallowing of thin and thick liquids (Fig. 2). The flow patterns varied with consistency of the liquid. Figure 3 shows the appearance of Annular flow during UES opening in thin liquid swallowing and thick liquid swallowing of every subject. Annular flow occurred in nearly every swallow of thin liquid across all subjects (3 ml 13/14 (93%), 10 ml 13/14 (93%), 20 ml 13/14 (93%)) but occurred in few swallows of thick liquid (3 ml 0/13 (0%), 10 ml 2/13 (15%), 20 ml 1/13 (8%) (Fig. 3). Plug flow was common in thick liquid. The percentage of swallows with Annular flow during the period of UES opening (sum of annular flow frames/number of frames that UES was open in all subjects) was significantly higher in swallows of thin liquid; 3 ml 41/71 (58%), 10 ml 49/85 (58%), 20 ml 49/88 (56%) than in swallows of thick liquid; 3 ml 0/64 (0%), 10 ml 3/78 (4%), 20 ml 1/80 (1%) (p < 0.01) (Fig. 4). In most cases, the onset of annular flow was during the second or third frames after onset of UES opening, and it ended one frame before UES closing (Fig. 3).

Detection of Annular flow or Plug flow had high interrater reliability for both thin and thick liquid swallows (Thin: Kappa = 0.940, Thick: Kappa = 0.855).

## Discussion

**Fig. 5:**
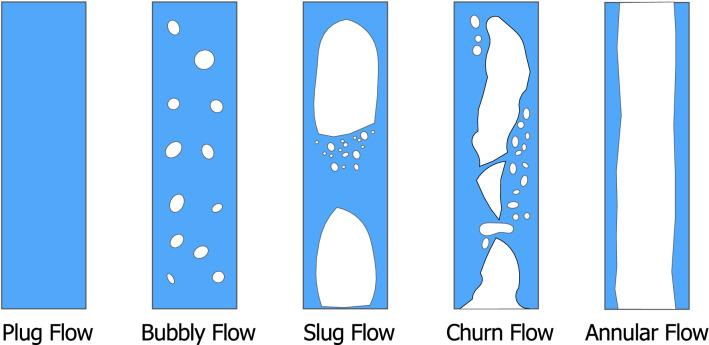
Typical flow patterns in vertical flow. Adapted from [9]. Blue is liquid, white is air

A liquid swallow is typically biphasic, including the liquid and also some air, so the actual swallowed bolus has both liquid and gas phases [7]. The patterns (regimes) of biphasic liquid and gas flow can be classified into four types: bubbly flow, slug flow, churn flow, and annular flow, depending on the balance of inertial, viscous, and frictional forces in the two phases (Fig. 5). Among them, Annular flow has a liquid layer on the inner wall of the lumen (channel) with the stream of gas in the center of the channel. Annular flow is typical for two-phase gas–liquid flow in a vertical or inclined channel. The liquid and gas phases typically have different velocities. Plug flow, on the other hand, is typical in swallows of a single liquid phase, or alternating between gas and liquid phases. It is assumed that there is no boundary layer adjacent to inner wall of the channel. Velocities are the same for the liquid and gas phases (at any given cross-section of the lumen). Two-phase gas–liquid flow has received extensive study for industrial applications, such as oil pipelines [7].

To our knowledge, this is the first report of annular flow in the UES during swallowing. The 3D dynamic CT allows easy viewing of cross sections at any angle. In axial sections, the ring of flowing liquid barium lined the wall of the UES and surrounded an inner core of moving air. In sagittal sections, continuous flow of liquid along with the wall of upper esophagus was observed as well as the continuous flow of air in the center of upper esophagus.

The pitfall of CT in terms of imaging is artifact [10]. In fact, in this study, we detected windmill artifact [10]. However, we considered that observed annular flow was not artifact because: 1. Windmill artifact was detected outside but not inside the esophagus. 2. If the observed phenomenon was motion artifact, this should be found in other phase of images when UES closed. However, this was not detected in other phases. 3. Bolus was flowing continuously through swallowing and thus, in the frame by frame analysis, the radiopaque (white) part was considered to be bolus, not artifact.

This study showed that annular flow was seen in most swallows of thin liquid, but was rarely seen in thick liquid swallows, supporting our hypothesis. Because the annular flow was observed in each bolus size with thin liquid and plug flow was observed in each bolus size with thick liquid, we infer that the difference in flow pattern between thin liquid–air and thick liquid–air boluses resulted from the differing magnitudes of viscous forces, not bolus size. A recent study quantifying the bolus flow for an in vitro model showed that oral transit time and bolus length were significantly longer in thinner (nectar thick) than in thicker (spoon thick) liquid regardless of the thickening agent [11]. When the UES opens, flow resistance decreases and the flow rate increases. With a biphasic thin liquid bolus, the liquid phase has higher viscous, cohesive and adhesive forces, than the air. This leads to the thin liquid adhering to the wall of the lumen. Because thick liquid has still higher vicosity, the magnitude of cohesive forces are greater, the thick liquid is not penetrated by the gas phase, leading to plug flow. Futhermore, because of the elongated thin liquid bolus, cross-sectional area is thought to be smaller. We infer that these flow patterns depend on the interaction of viscosity with cohesion and adhesion.

Another variable of interest is the amount of air in the pharynx at the time of the swallow. In this study, we did not measure pharyngeal volume. However, a recent study from our group showed that pharyngeal volume was usually higher for thin than for thick liquids at the onset of UES opening [12]. This can be observed in the lateral images in Fig. 2 (of the present paper) where the pharyngeal cavity was larger in swallow of thin liquid than thick liquid at 0.1 s. When the UES opened, the larynx and velopharynx were closed, and thus, air in pharynx could only be discharged through UES to esophagus. This suggests that larger amount of air is swallowed with thin liquids. Another previous study reported that pharyngeal volume was significantly higher at the onset of UES opening for larger volume of bolus [13]. Thus, the amount of air swallowed might increase as the bolus volume increases. Further study is necessary to address this point by measuring the pharyngeal volume of air in different bolus consistencies and bolus volumes.

Walczak et al. reported that intrabolus pressure (IBP) changed with the location in the pharynx and location along axis of flow of the bolus (bolus head, mid-bolus, bolus tail) in the study of IBP using high-resolution manometry [14]. They reported the mid-bolus pressure was highest at the tongue base, decreased at the hypopharynx, and reached its minimum at the distal UES, where it was lower than leading bolus pressure. They explained that lower IBP at UES was associated with the volume of bolus substance. The current finding of annular flow may explain the decrease of mid-bolus IBP pressure at the diatal UES in their study, since the manometer could have been in the gas phase of the bolus rather than the liquid. Observing the bolus flow pattern in cross-section can provide a new window into the understanding of intrabolus pressure and flow.

Annular flow is rarely observed in two-dimensional swallow studies such as videofluoroscopy. This is mainly because the transverse view is not available in videofluoroscopy. Also, because of the fluoro images, even if the annulus of barium is lining the wall of esophagus around an inner core of air, the lumen appears to be filled with the barium. It suggests that the area of barium may not correlate with the actual volume of bolus in the pharynx and esophagus in videofluoroscopy [15].

A few limitations of this study should be noted. First, because of the radiation dose, we could not compare the two bolus consistencies within the same subject. We do not believe this obviates our result that annular flow was strongly associated with thin liquid-swallows. In the future studies, by comparing the different consistencies within the same subject, the effect of UES size and shape on the bolus flow through UES can be explored. Second, the scanning was performed in semi-reclining posture, since the upright posture was not possible with three-dimensional CT scanning. The effect of gravity in this position could conceivably have influenced the bolus flow patterns we observed. The semi-reclining posture was consistent with all swallows across all subjects; thus, it seems unlikely that this could explain the observed differences between consistencies. Lastly, the rheology of the bolus flow was not analyzed mathematically or hydrodynamically. Future studies using those approaches might reveal detailed mechanisms of the gas–liquid flow pattern and the effects of consistency more clearly. The study to construct swallowing simulation by smoothed particle hydrodynamics using 3D dynamic CT data has been started, for example [16].

In conclusion, our results revealed different bolus flow patterns through UES in thin liquid–air and thick liquid–air swallows. Annular flow was seen during UES opening in most thin liquid–air swallows. In contrast, Plug flow was seen in nearly all thick liquid swallows. These difference may result from the rheologic properties of the bolus including the interaction of viscosity with cohesion and adhesion. Differing air volumes in the pharynx could also influence the patterns of bolus flow; this warrants further study.
