# Correction to: Annular Flow in the Upper Esophageal Sphincter Demonstrated with Dynamic 320-row Area Detector Computed Tomography

**DOI:** 10.1007/s00455-021-10282-8

**Published:** 2021-03-15

**Authors:** Yoko Inamoto, Eiichi Saitoh, Jefrey B. Palmer

**Affiliations:** 1 Faculty of Rehabilitation, School of Health Sciences, Fujita Health University, 1‑98 Dengakugakubo, Kutsukake, Toyoake, Aichi 470‑1192 Japan; 2 Department of Rehabilitation Medicine I, School of Medicine, Fujita Health University, Toyoake, Aichi Japan; 3 Department of Physical Medicine and Rehabilitation, Johns Hopkins University, Baltimore, MD USA

## Correction to: Dysphagia 10.1007/s00455-020-10241-9

The article “Annular Flow in the Upper Esophageal Sphincter Demonstrated with Dynamic 320-row Area Detector Computed Tomography”, written by “Yoko Inamoto, Eiichi Saitoh, Jefrey B. Palmer” was originally published electronically on the publisher’s internet portal on 28 January 2021 without open access. With the author(s)’ decision to opt for Open Choice the copyright of the article changed on 1 March 2021 to © The Author(s) 2021 and the article is forthwith distributed under a Creative Commons Attribution 4.0 International License, which permits use, sharing, adaptation, distribution and reproduction in any medium or format, as long as you give appropriate credit to the original author(s) and the source, provide a link to the Creative Commons licence, and indicate if changes were made. The images or other third party material in this article are included in the article’s Creative Commons licence, unless indicated otherwise in a credit line to the material. If material is not included in the article’s Creative Commons licence and your intended use is not permitted by statutory regulation or exceeds the permitted use, you will need to obtain permission directly from the copyright holder. To view a copy of this licence, visit http://creativecommons.org/licenses/by/4.0

The original article has been corrected.

